# Cost savings and quality-of-life gains associated with the use of an AI-powered digital tool for self-management of chronic pain based on a multicenter cohort study

**DOI:** 10.1093/pm/pnag061

**Published:** 2026-05-04

**Authors:** Sara Olofsson, Maria L Rosén Klement, Antje M Barreveld, Neel Mehta, Ulf Persson, Carl A K Borrebaeck

**Affiliations:** The Swedish Institute for Health Economics (IHE), Lund, Sweden; Department of Immunotechnology, Lund University, Medicon Village, Lund, Sweden; Department of Anesthesiology, Tufts University School of Medicine, Newton-Wellesley Hospital, Newton, MA, United States; Department of Anesthesiology, Division of Pain Management, Weill Cornell Medicine, New York, NY, United States; The Swedish Institute for Health Economics (IHE), Lund, Sweden; Department of Immunotechnology, Lund University, Medicon Village, Lund, Sweden

**Keywords:** chronic pain, costs, quality of life, digital, artificial intelligence, self-management

## Abstract

**Background:**

Chronic pain is a highly prevalent disease, leading to high health care costs, impaired productivity, and decreased quality of life. An artificial intelligence (AI)–powered digital tool for self-management (Paindrainer^TM^) has been shown to be an effective treatment in chronic pain and could have potential to reduce the burden on costs and quality of life.

**Objective:**

The objective of this study was to estimate the cost savings and quality-of-life gains associated with the use of an AI-powered digital tool for self-management of chronic pain. The study is a subsequent health economic analysis of previously published data.

**Method:**

Resource use and quality-of-life data were derived from a 1-arm, multicenter study in the United States. Differences between baseline and follow-up (6 and 12 weeks) were translated to an annual monetary estimate by using published literature, national statistics, and accepted threshold values for a quality-adjusted life year (QALY).

**Result:**

At 12 weeks, the use of an AI-powered digital tool was associated with a reduction in health care costs of $127 per user and an increase in annual productivity of $930 per user. In addition, the intervention was associated with an increase in health of 0.0275 QALYs, corresponding to a monetized value of $1375.

**Conclusion:**

The analysis of cost savings and quality-of-life gains associated with the use of an AI-powered digital tool for pain self-management demonstrates significant potential improvements in measured parameters of up to $2432 per person in total ($7380 per year, assuming 70% compliance), supporting this as an important treatment alternative.

## Teaser

The objective of this study was to estimate the cost savings and quality-of-life gains associated with the use of an AI-powered, digital tool for self-management of chronic pain. Resource use and quality-of-life data was derived from a one-arm, multicenter cohort study in the US. After 12 weeks, health care costs per person were reduced by $127 and productivity increased by $930. In addition, the intervention resulted in a gain of 0.0275 QALYs.

Article Summary

- The objective of this study was to estimate the cost savings and quality-of-life gains associated with the use of an AI-powered digital tool for self-management of chronic pain.
- Resource use and quality-of-life data were derived from a 1-arm, multicenter cohort study in the United States.
- After 12 weeks, health care costs per person were reduced by $127, and productivity increased by $930. In addition, the intervention resulted in a gain of 0.0275 QALYs.

## Introduction

Chronic pain is a highly prevalent disease that affects approximately 1 in 4 US adults^1^ and contributes to a significant burden on individuals, relatives, employers, and health care payers. Gaskin and Richard found that the total financial cost of pain in the United States, in terms of incremental health care cost and productivity losses, was $560–635 billion in 2010 dollars—greater than the annual costs of heart disease ($309 billion), cancer ($243 billion), or diabetes ($188 billion).^2^ The health care cost of chronic pain has increased over time, partly because of changes in pain medication prescribing practices. On the basis of claims data from 2022, Schoenfeld et al. estimated the annual, all-cause health care cost for individuals with chronic pain in the United States at $725 billion.^3^ The cost of chronic pain has also been found to be substantial in register data from other countries such as Sweden^4^ and Norway,^5^ amounting to between 4% and 10% of gross domestic product (GDP). Furthermore, Gaskin and Richard reported that medical expenditures for chronic pain in the United States increase rapidly by pain severity (around twice as high for severe as for moderate pain).^2^ A similar trend was also revealed when health care resource use was compared between US individuals with high-impact chronic pain and individuals with chronic pain without limitations in a study by Pitcher et al. based on the 2011 National Health Interview Survey.^6^

In addition to the significant cost burden, which to a large extent is carried by health care payers and employers, chronic pain imposes a heavy burden on individuals in terms of its impact on health-related quality of life (HRQoL). Hogan et al. (2017) found a mean utility of 0.59 (on a scale from 0 [death] to 1 [full health]) among a large cohort with chronic pain (*n* = 12 146) in the Canadian Community Health Survey (CCHS), which is lower than what is observed in other chronic diseases, such as heart disease and diabetes.^7^ A similar utility (0.58) was reported by Wayne et al. for a US sample of patients (*n* = 144) with chronic low back pain.^8^

Managing chronic pain is complex because of the involvement of multiple interconnected factors. Physical pain signals and neurological changes interact with psychological aspects such as anxiety and depression, as well as sleep disturbances and progressive loss of function in daily activities. These symptoms are highly individual, requiring treatment to be tailored to each person’s unique pain pattern, psychological state, social circumstances, and physical limitations.^9^

Traditional biomedical approaches addressing only physical symptoms have shown limited efficacy for chronic pain, as both pain perception and associated disability are strongly influenced by psychological and social factors. This limitation has led to the adoption of biopsychosocial frameworks that recognize pain as a multidimensional experience requiring integrated intervention. Multidisciplinary approaches combining physical therapy, medication management, social support, and psychological components are typically recommended. Patient-centered care is essential within this framework, as pain experiences, functional limitations, and treatment responses vary significantly across individuals, necessitating tailored intervention strategies that align with each person’s specific needs, values, and life context.^9^^,^^10^

Psychological interventions form a critical component of comprehensive pain management. Cognitive behavioral therapy (CBT) and acceptance and commitment therapy (ACT) are commonly used as part of multidisciplinary pain management programs. ACT is grounded in relational frame theory and centers on psychological flexibility. This refers to the ability to remain present with one’s experience and take value-guided action, even when pain limits previous approaches to valued activities. The therapy operates through 6 core processes: acceptance (willingness to experience pain without struggle), cognitive defusion (reducing the impact of unhelpful thoughts about pain), present moment awareness (mindful attention to current experience), self-as-context (observing thoughts and feelings from a stable sense of self), values clarification (identifying what matters most in life), and committed action (taking steps toward valued goals through flexible approaches).^11^ A key aspect of ACT involves helping individuals distinguish between specific activities and their underlying core values, and then finding alternative ways to honor those values when pain makes the original activities difficult or impossible. This approach emphasizes behavioral flexibility—adapting how we engage with what matters most rather than abandoning valued life domains entirely because of pain limitations. The focus shifts from maintaining exact previous behaviors to preserving the essential meaning and purpose behind those activities through modified or alternative approaches.

Meta-analytic evidence supports moderate to large effect sizes (*d* = 0.5–0.7) for ACT-based interventions in reducing pain interference and improving functional outcomes, with therapeutic benefits typically sustained across 6 to 12 months, which suggests the potential for durable clinical impact.^12^ These treatments can be costly, as they typically require personal guidance by health care providers on a regular basis, and effects have been found to decrease over time after guidance is removed.^13^ Access to these treatments might also be restricted by limited insurance or availability of specialty care.

Digital self-guided treatments could provide a cost-effective way of implementing effective pain treatment, with several important advantages, including lower cost for health care payers, sustained effect for the individual because of the possibility of continued digital coaching, and increased accessibility.^14^^,^^15^ Artificial intelligence (AI) could advance personalized health care by delivering individualized treatments tailored to each patient’s unique needs, such as progressively adjusting activity levels according to the patient’s tolerance and activity-to-recovery time ratio. AI simultaneously reduces cognitive burden through automated decision support, real-time monitoring, and streamlined self-management tools that simplify complex treatment protocols. Additionally, AI provides personalized behavior change guidance by analyzing individual patient patterns, identifying barriers to adherence, and delivering timely, contextual interventions that support sustainable lifestyle modifications.^16^ A prospective, 12-week, multicenter, single-arm clinical study using an AI-powered, patient-centric digital tool for self-management of chronic pain (Paindrainer^TM^) showed a significant improvement in minimal important difference (MID) for physical function measured with the Patient-Reported Outcomes Measurement Information System (PROMIS®) for almost 75% of patients (*n* = 41).^10^

To provide payers and policymakers with relevant information, it is important to demonstrate the value of new interventions. In the context of chronic pain, impacts outside of health care are likely to be substantial, and it is therefore important to move beyond the health care perspective. Traditional value components from a societal perspective include health care costs but also gains in capacity to work (productivity) and quality of life. The objective of the present study is to estimate impacts on costs and HRQoL associated with the use of an AI-powered digital tool for self-management of chronic pain to provide an estimate of the total potential value from a societal perspective.

## Methods

### Design

This study was based on data from an open, single-arm, multicenter clinical trial (Paindrainer trial) performed at Newton-Wellesley Hospital (NWH) and NewYork-Presbyterian Weill Cornell Medical Center (WCMC) in New York City. The details of the trial are presented in Barreveld et al. (2023).^10^ Because the study did not include a control group, strict inclusion criteria were used, and the baseline measures were used to estimate any effect of the intervention at 6 and 12 weeks. All subjects continued standard treatment and rehabilitation for their conditions (eg, physical therapy, medications, interventions, education in pain management strategies), as applicable.

### Participants

The participants in the trial were adult individuals with lower back or neck pain of at least 3 months’ duration and a pain score of at least 4 on the numeric rating scale. Ninety-four individuals consented to participate in the study, of whom 43 individuals responded to the follow-up survey at 6 weeks and 34 individuals responded to the follow-up survey at 12 weeks.

### Intervention

The intervention was an AI-powered digital tool (Paindrainer^TM^) for self-management of chronic pain based on the principles of ACT and standard clinical practice. This means that it incorporates evidence-based approaches typically used in clinical pain management, such as pacing, progressive enhancement of physical activity levels, and promotion of healthy sleep patterns. The AI engine coaches each individual patient on the basis of his or her own data input. The individual first logs activities and pain levels for 8 days, and the AI-engine then creates an individualized coaching plan to reach an activity balance with an acceptable level of pain.

### Outcomes

The outcomes of this study include health care use and medications (direct medical costs), production loss (indirect cost), and HRQoL. Data were derived from the clinical trial at baseline and at 6 and 12 weeks of follow-up. Data on health care use, medications, and ability to work were available for only 1 center (NWH), whereas HRQoL data were available for both centers.

### Statistical analysis

Use of health care services and medications was reported at each time point, but the amount of health care or medication used was not reported. To estimate direct medical costs, the share of respondents with treatment at each time point was estimated, with the number of respondents with a 6-week follow-up used as a fixed denominator. The change in the share with treatment and medication utilization was then translated to a monetary estimate by multiplying it with the average cost for office visits ($1279 per year) and medication ($2656 per year) for usual care, as reported in a randomized controlled trial performed at one of the centers included in the Paindrainer trial, the Brigham and Women’s Hospital, and commensurate with other large academic medical centers.^8^

Data on the mean daily capacity to work were used to estimate indirect costs. The change in capacity to work was translated to a monetary estimate by using the human capital approach,^17^ assuming that the value of production corresponds to what the employer pays for the employee, ie, gross wage including payroll taxes:

- According to the Current Population Survey (CPS), the mean earnings per week in the first quarter of 2023 was $1100.^18^
- Assuming the typical work week is 40 hours, this would correspond to $27.5 per hour ($1100 / 40 hours).
- Adding 15% of payroll taxes^19^ results in a total value of $31 per hour (1.15×$27.5).
- The annual gain is estimated by the number of workdays in a year, which was estimated to 240 after subtracting weekends (104), bank holidays (10),^20^ and vacation days (11)^21^ from 365 days.

HRQoL was measured in the trial via PROMIS®, a health profile measurement system developed by the National Institutes of Health (NIH), which includes item banks for several different domains (eg, pain, physical function, sleep, social activity).^22^ The Paindrainer trial included the domains of pain interference, pain intensity, physical function, depression, and anxiety. Utility scores (preference-based measurement of health from 0 = death to 1 = full health) were calculated by using the value set of Craig et al. (2021)^23^ developed for PROMIS-29, a questionnaire generated for measurement of several domains. As items differed slightly from the domains in the trial, some assumptions and simplifications were applied (see Supplementary Material). To validate results, utility scores were also estimated from the value set of Dewitt et al. (2018)^24^ and compared with the utility scores estimated from Craig et al. (2021) (see Supplementary Material). The HRQoL gain was translated to a monetary estimate by multiplying it by a typically accepted threshold for a QALY of $50 000.^25^

Statistically significant differences in resource use (health care, medication), work capacity, and HRQoL at 6 and 12 weeks (vs baseline) were estimated with *t* tests, with a *P* value <.05 considered statistically significant. The monetary value was estimated for each component at 12 weeks and summarized to estimate total value. In a sensitivity analysis, gains were assumed to continue for the remaining year for 70% of users. A compliance rate of 70% is based on the observation that 9 of 43 individuals at the 6-week follow up (21%) did not respond to the 12-week follow-up, as well as a study reporting compliance with pain rehabilitation beyond 6 months at 70%.^26^

## Results

### Health care resource use

At baseline, a majority of subjects were receiving chronic pain treatment (80.6%, *n* = 25) and taking medications for pain (83.9%, *n* = 26) (Table 1). The number of respondents with any treatment was reduced from baseline by 12% (67.7%, *n* = 21) at 6 weeks and by 23% (58.1%, *n* = 18) at 12 weeks. The reduction was seen primarily in the use of physical therapy, CBT, and chiropractor. The number of respondents with pain and mental health medication was reduced by 10% at 6 and 12 weeks of follow-up (74.2%, *n* = 23). The reduction was seen primarily in the use of nonsteroidal anti-inflammatory drugs and antidepressants for mood.

**Table 1:** Health care resource use for participants at the Newton-Wellesley Hospital (NWH) and estimated health care cost savings.

Resource use	Baseline *n* = 31	6 weeks *n* = 31	6 weeks vs baseline	12 weeks *n* = 31	12 weeks vs baseline	12 weeks vs 6 weeks
**Physical therapy**	54.8% *n* = 17	41.9% *n* = 13		25.8% *n* = 8		
**Injection therapy**	25.8% *n* = 8	25.8% *n* = 8		29.0% *n* = 9		
**Cognitive behavioral therapy**	12.9% *n* = 4	9.7% *n* = 3		6.5% *n* = 2		
**Mindfulness meditation course**	3.2% *n* = 1	9.7% *n* = 3		9.7% *n* = 3		
**Regular home mindfulness/relaxation practice**	29.0% *n* = 9	25.8% *n* = 8		25.8% *n* = 8		
**Acupuncture**	6.5% *n* = 2	9.7% *n* = 3		6.5% *n* = 2		
**Chiropractor**	16.1% *n* = 5	12.9% *n* = 4		6.5% *n* = 2		
**Any treatment**	**80.6%** ***n* = 25**	**67.7%** ***n* = 21**	**−12.9%** ***P* = .1607** ***n* = 31**	**58.1%** ***n* = 18**	**−22.6%** ***P* = .0034** ***n* = 31**	**−9.7%** ***P* = .2636** ***n* = 31**
**Nonsteroidal anti-inflammatory drugs (NSAIDs, eg, ibuprofen/Motrin, naproxen, celecoxib/Celebrex etc.)**	67.7% *n* = 21	35.5% *n* = 11		45.2% *n* = 23		
**Acetaminophen (Tylenol)**	25.8% *n* = 8	45.2% *n* = 14		38.7% *n* = 12		
**Nerve medication (eg, gabapentin, pregabalin, etc.)**	22.6% *n* = 7	19.4% *n* = 6		25.8% *n* = 8		
**Antidepressants for mood**	6.5% *n* = 2	6.5% *n* = 2		3.2% *n* = 1		
**Antidepressants for pain (eg, amitriptyline, nortriptyline, duloxetine, etc.)**	12.9% *n* = 4	19.4% *n* = 6		16.1% *n* = 5		
**Antianxiety medications on a regular basis (eg, benzodiazepines such as lorazepram or clonazepam)**	9.7% *n* = 3	12.9% *n* = 4		9.7% *n* = 3		
**Muscle relaxants (eg, cyclobenzaprine, Flexenil, tizanidine, etc.)**	32.3% *n* = 10	32.3% *n* = 10		32.3% *n* = 10		
**Any medication**	**83.9% *n* = 26**	**74.2% *n* = 23**	**−9.7% *P* = .1849 *n* = 31**	**74.2% *n* = 23**	**−9.7% *P* = .2636 *n* = 31**	**-**

Differences tested with *t* test.

### Production gain

At baseline, the daily capacity to work was 371 minutes on average (SD: 149) (Table 2). At 6 weeks of follow-up, the daily capacity to work had increased by 26.1 minutes on average (SD: 89.8, range −105 to +237) compared with baseline. At 12 weeks of follow-up, the increase in daily capacity to work had increased by 32.6 minutes (SD: 100, range −132 to +322) compared with baseline.

**Table 2:** Mean daily capacity to work in minutes (standard deviation) for employed participants and estimated monetary production gain per year.

	Baseline	6 weeks	*6 weeks vs baseline*	12 weeks	*12 weeks vs baseline*	*12 weeks vs 6 weeks*
**Capacity to work in minutes per day**	371 (149) *n* = 31	397 (149) *n* = 31	*26.1 P* *=* *.1166 n* = *31*	389 (162) *n* = 26	*32.6 P* *=* *.1105 n* = *26*	*3.35 P* *=* *.7083 n* = *26*

Differences tested with *t* test.

### Health-related quality of life

At baseline, the HRQoL was 0.424 (Table 3)—that is, 0.576 below full health (1.0). The main domain impacting HRQoL was physical function (−0.30), followed by pain interference (−0.11), anxiety (−0.096), and depression (−0.065). At 6 weeks, there was an average increase in HRQoL of 0.170. A significant improvement was seen in all domains except physical function. At 12 weeks, the average increase in HRQoL compared with baseline was 0.119. The increases in HRQoL in the domains of anxiety and pain interference were slightly reduced compared with those at 6 weeks. The QALYs gained at 12 weeks were estimated by multiplying the HRQoL (0.119) by the time in years (12 weeks/52 weeks), resulting in 0.0275 QALYs.

**Table 3:** Mean utilities (standard deviation) and estimated QALY gain based on PROMIS translated with Craig et al. (2021).

Dimension	Baseline	6 weeks	*6 weeks vs baseline*	12 weeks	*12 weeks vs baseline*	*12 weeks vs 6 weeks*
**Anxiety**	−0.096 (0.159) *n* = 45	−0.051 (0.094) *n* = 42	***0.049*** ***P*** ***=*** ***.0245*** ***n* = *42***	−0.058 (0.121) *n* = 35	*0.0154* *P* *=* *.313* *n* = *35*	*−0.012* *P* *=* *.2615* *n* = *35*
**Depression**	−0.065 (0.109) *n* = 45	−0.031 (0.065) *n* = 42	***0.037*** ***P*** ***=*** ***.0048*** ***n* = *42***	−0.028 (0.062) *n* = 34	***0.038*** ***P*** ***=*** ***.0141*** ***n* = *34***	*0.002* *P* *=* *.8577* *n* = *34*
**Pain interference**	−0.110 (0.120) *n* = 44	−0.084 (0.112) *n* = 42	***0.031*** ***P*** ***=*** ***.0460*** ***n* = *41***	−0.087 (0.113) *n* = 35	*0.016* *P* *=* *.4589* *n* = *34*	*−0.013* *P* *=* *.4281* *n* = *35*
**Physical function**	−0.300 (0.404) *n* = 45	−0.261 (0.406) *n* = 42	*0.055 P* *=* *.1440 n* = *42*	−0.269 (0.428) *n* = 35	*0.056 P* *=* *.3410 n* = *35*	*0.000 P* *=* *.9984 n* = *35*
**TOTAL**	−0.576 (0.563) *n* = 44	−0.427 (0.539) *n* = 42	***0.170 P*** ***=*** ***.0046 n* = *41***	−0.452 (0.563) *n* = 34	*0.119 P* *=* *.1624 n* = *33*	*−0.024 P* *=* *.7365 n* = *34*
**QALYs^a^**			0.0196		0.0275	

Differences tested with *t* test. Significant results (*p* < 0.05) is included as bold text.

a Utility × number of weeks / 52 weeks for a year.

### The monetary value of changes associated with the use of an AI-powered digital tool for self-management of chronic pain

The estimated reduction in health care resource use at 12 weeks would translate to a health care cost savings of $127 per user ($68 for office visits and $59 for medication) given the assumptions applied (see the “Statistical analysis” section). In addition, the increase in productivity corresponds to a monetary value of $930 per user. Finally, the increase in health (0.0275 QALYs) could be translated to a monetary value of $1375 by applying the common threshold value of $50 000. In total, the use of an AI-powered digital tool for self-management of chronic pain is associated with a monetary value of $2432 per user. Under the assumption of 70% compliance^26^ and no waning effect, the annual monetary value would correspond to $7380 per user (Table 4).

**Table 4:** Estimated monetary value of changes from baseline to 12 weeks.

Type of cost	Change from baseline to 12 weeks	Unit cost/value	Monetary value of change at 12 weeks	Extrapolated monetary value of change at 52 weeks assuming 70% compliance^e^
**Health care**				
** Office visits**	−22.6% (Table 1)**^a^**	$25/week^8^	$68**^b^**	$200
** Medication**	−9.7% (Table 1)^a^	$51/week^8^	$59^b^	$180
**Productivity**	+32.6 min/day (Table 2)	$31/hour (“Statistical analysis” section)	$930^c^	$2800
**Health**	+0.0275 QALYs (Table 3)	$50 000/QALY^25^	$1375^d^	$4200
**TOTAL**			**$2432**	**$7380**

a Reduction in share of users with any health care visits or any medication. Assumed to correspond to the share in reduction of health care cost estimated in Wayne et al. (2019).^8^

b Reduction in share of users × unit cost × 12 weeks.

c Increase in minutes per day / 60 minutes per hour × unit cost× 12 weeks × 240 workdays / 365 calendar days.

d 12 weeks / 52 weeks in a year × unit value.

e 52 weeks / 12 weeks × monetary value of change at 12 weeks× 70% (rounded numbers).

## Discussion

This study has estimated that the use of an AI-powered digital tool for self-management of chronic pain is associated with a reduction in health care costs at 12 weeks of $127 per person and an increase in productivity at 12 weeks of $930 per person. In addition, the intervention was associated with an estimated increase of 0.0275 QALYs at 12 weeks, corresponding to a monetized value of $1375. The total value of changes associated with the intervention was $2432, which would correspond to an annual value of around $7380 per person based on the assumed compliance of 70%.

A systematic review of the cost-effectiveness of a set of diverse pain management services (defined as health services targeting at least 2 of the social, physical, psychological, and occupational aspects provided by 1 or more health care professionals) for chronic low back pain found that only 1 of 5 studies (integrated care vs usual care in the Netherlands) resulted in a favorable cost-effectiveness, with cost savings and improved HRQoL (ie, dominant).^27^ Other treatments were usually cost-saving but less effective (intensive rehabilitation vs surgery in the UK, multidisciplinary intervention vs total disc replacement in Norway, combined treatment vs active physical treatment) or both cost-increasing and less effective (combined treatment vs graded activity plus problem-solving).^17^ A narrative review of multidisciplinary interventions for chronic low back pain also found that only 2 of 7 studies presented favorable cost-effectiveness.^28^ A US study of the cost-effectiveness of team-based integrative medicine at an integrative medicine clinic in an academic medical center for chronic low back pain (*n* = 134) showed that costs were higher compared with usual care at other clinics at the same hospital (*n* = 144) and that QALYs gained did not differ after adjustment for baseline differences.^8^ In comparison, the result of the present study appears promising, showing significant potential cost savings as well as QALY gains.

Traditional pain management programs face significant cost-effectiveness challenges, where multidisciplinary intervention is reported to cost around €6000 (∼$7000) per person because of the high frequency of health care visits.^27^ Digital health solutions, including mobile applications and web-based self-management platforms, offer a pathway to both expand access and reduce costs. These interventions minimize dependence on health care resources and overcome barriers related to geography, mobility, and appointment availability, enabling cost-effective delivery of evidence-based care.

Two randomized controlled trials showed that self-guided pain management services have the potential to reduce health care costs without compromising effectiveness. Paganini et al. (2019) assessed costs and effects of an Internet- and mobile-based intervention (ACTonPain) with different levels of clinical support for adults with chronic pain in Germany (*n* = 302). The results showed that both the unguided and the guided versions resulted in improved HRQoL compared with the control group (waitlist). However, whereas the unguided version also resulted in lower costs (−€342 per year), the guided version resulted in higher costs (+€660 per year) compared with a waitlist control group. Dear et al. (2021) estimated the costs and effects of an Internet-delivered pain management program (Pain Course) delivered with different levels of clinical support for individuals with chronic pain (*n* = 490) in Australia. They also found that both the unguided and the guided versions led to improvements compared with the waitlist group, but the unguided version resulted in larger cost savings when compared with controls. These findings have important implications for AI-powered digital interventions, which can provide real-time, individualized activity planning that adapts to daily pain patterns. The integration of AI for personalized pain management thus represents an emerging area. Future comparative-effectiveness studies should investigate whether AI-powered digital tools deliver additional health economic benefits beyond those provided by traditional digital interventions.

The clinical effectiveness underlying this health economic calculation exceeds that reported in the broader literature. Although meta-analytic evidence indicates modest effect sizes (SMD 0.25–0.37) for digital pain interventions,^29^ the present AI-powered intervention achieved substantially higher rates of clinically meaningful improvement (72% to 100% across validated domains), which suggests greater efficacy for a personalized AI approach.

The present study is based on self-reported data from a 12-week prospective, multicenter clinical trial. Self-reported data can be inaccurate, but self-reporting is often used as a pragmatic approach to collect data on resource use and work capacity, and it is the only possible way of collecting data on quality of life. The data are based on a limited sample size, and data on health care resource use were available from only 1 clinic. The results should therefore be interpreted with caution and might not be generalizable to other clinics.

The cost savings and changes in HRQoL were estimated from the measurement at 12 weeks. Thus, it is assumed that the changes at this point in time could be sustained for 3 months. It should be noted that these estimations do not represent the actual changes seen during the 12 weeks of the trial, as these varied over time (see changes between 6 and 12 weeks). The changes reported during the course of a trial would, however, not necessarily correspond to changes over time in the real world. The clinical trial covers a period during which treatment is initiated and has a protocol-driven end. In the real world, treatment continues for some users and ends for others. Estimating changes limited to the time frame of the clinical trial most likely means an underestimation, as it does not consider users who continue to benefit from the intervention. On the other hand, extrapolating to the entire year is likely to cause overestimation, as it ignores users who stop using the tool or experience a waning effect. A future study based on real-world data could aim to adjust for this by taking these factors into consideration.

The clinical trial is an open, 1-arm trial—that is, a comparison of before and after the intervention. In the absence of a control group, it was assumed that users would have remained at baseline without the intervention. The users were monitored for change of care, additional medication, and nerve blockades during the intervention period, which otherwise could have affected the outcome. It is plausible that participants of a potential control group (individuals with chronic pain and without intervention) might have received additional care to manage their pain. If this were the case, the estimated cost offsets would be underestimated, as would be the value of the AI-powered digital tool treatment. This was the case in Dear et al (2021),^14^ which showed that the waitlist control group reported an increase in health care cost over time.

The trial reported only the share of users stopping treatment or medication. There could also be users who reduced their number of visits or their medication dose. As this was not included in the calculations, the results should be regarded as conservative. Moreover, there is often a delay in the reduction of health care costs, as users might be on a preset treatment schedule. A long-term follow-up study of both discontinuation and reduction of treatment and medication could provide more appropriate data for calculation of health care cost savings. The reduction in health care costs reported in this study ($127 for 12 weeks, or around $480 for a year) are in line with health care savings as reported by the randomized controlled trials of unguided pain management programs ($397–$504 for a year^14^^,^^15^).

Pain-related productivity loss exerts a substantial economic burden, with annual costs of up to $335 billion in the United States, exceeding direct health care expenditures for musculoskeletal pain.^2^^,^^30^ Production gain was estimated from the daily capacity to work, as reported by the users enrolled in the clinical trial. This type of data is unique and allows for an analysis of the real performance at work. However, it is not clear what the gain in daily capacity to work represents. Production gain can be a consequence of individuals being able to reduce their absence from work (absenteeism) or being able to work more while present at work (presenteeism). Studies of productivity among individuals with chronic pain suggest that absenteeism, in terms of sick leave, is limited among individuals with chronic pain.^2^^,^^8^^,^^31^ However, absenteeism, in terms of making a choice to work fewer hours,^2^ is more common, as is presenteeism.^31^ The increase in productivity reported in the present study ($930 for 12 weeks, or around $2800 for a year) is roughly in line with the production gain reported for an unguided pain management program in Germany by Paganini et al. (2019) ($2021 for a year)^15^ and the $2916 annual savings reported by Janela et al. for a multimodal digital care program in the United States.^32^ It should, however, be noted that the increase in productivity could differ across populations and settings, and the generalizability of the result might be limited.

Health gain was estimated in terms of the number of QALYs gained based on quality of life measured by PROMIS. Two different approaches were used to translate the data from the clinical trial to preference-based quality-of-life weights. Both approaches resulted in similar results (see Supplementary Material). The increase in QALYs in this study (0.0275 QALYs for 12 weeks, or around 0.08 for a year) could be compared with the QALY gain reported for multimodal pain rehabilitation (0.097).^33^

The significant burden (eg, very low quality of life) of chronic pain and the few available treatments that have been found to be cost-effective suggest that there is an unmet need in the treatment of chronic pain. Thus, there is a need for effective and cost-saving treatment alternatives, such as an AI-powered digital tool for self-management of chronic pain. These data were derived from a 1-arm trial and were found to report potential improvements in all health economic outcomes. Future research could help validate the findings by including a control group to assess outcomes in comparison with treatment alternatives, longer-term follow-up to assess compliance, and subgroup analysis to assess who is likely to benefit most from the intervention.

## Conclusion

Traditional pain management programs face significant cost-effectiveness and access challenges. Digital health solutions, including mobile applications and web-based self-management platforms, offer a pathway to both expand access and reduce costs. These interventions minimize dependence on health care resources and overcome barriers related to geography, mobility, and appointment availability, enabling cost-effective delivery of evidence-based care.

This study shows that the use of an AI-powered digital tool for self-management of chronic pain (Paindrainer^TM^) is associated with a reduction in health care costs and increases in productivity and HRQoL. Implementing this tool can therefore be expected to have the potential to assist in relieving the substantial burden of chronic pain. Compared with other pain management programs, where few present favorable cost-effectiveness, the results appear promising. Areas for future research could include relative-effectiveness studies to investigate the gains in direct comparison with other treatments and subgroup analyses to assess which individuals benefit most from the intervention.

## Supplementary Material

pnag061_Supplementary_Data
