# Supplementary material for: Cost savings and quality-of-life gains associated with the use of an AI-powered digital tool for self-management of chronic pain based on a multicenter cohort study
Source: Pain Med. 2026 May 4;27(9):1056–63. doi: 10.1093/pm/pnag061 (PMC13537735; doi:10.1093/pm/pnag061)
Supplement: Supplementary file 1 [file pnag061_supplementary_data.docx]

**Supplementary material**

**Table S1**. Items in the PROMIS instruments used in the clinical trial and in the PROMIS-29 value set (grey rows = corresponding items used for translating clinical trial data to preference-based quality of life using Craig et al.)

| Domain | Items in PROMIS used in the clinical trial | Items used in PROMIS-29 value set |
| --- | --- | --- |
| Physical function | Vigorous activities |  |
|  | Walking – limit more than a mile | Walking – at least 15 minutes |
|  | Stairs – limit in climbing one flight of stairs | Stairs – up and down, normal pace |
|  | Lifting/Carrying groceries | Run errands and shop |
|  | Bending, kneeling, stooping |  |
|  | Chores – vacuuming, yard work | Chores – vacuuming, yard work |
|  | Dress yourself |  |
|  | Shampoo hair |  |
|  | Wash and dry body |  |
|  | Sit on and get up from toilet |  |
|  |  |  |
| Pain interference | Day-to-day activities | Day-to-day activities |
|  | Work around home | Work around the home |
|  | Social activities | Social activities |
|  | Household chores |  |
|  | Things do for fun |  |
|  | Enjoyment of social activities | Enjoyment of social activities |
|  |  |  |
| Depression | Worthless | Worthless |
|  | Helpless | Helpless |
|  | Depressed | Depressed |
|  | Hopeless | Hopeless |
|  |  |  |
| Anxiety | Fearful | Fearful |
|  | Focus | Focus |
|  | Overwhelmed by worries | Overwhelmed by worries |
|  | Uneasy | Uneasy |

Utility gain from baseline

|  | PF, PI, DP, AX (Craig et al.) | PF, PI, DP (Craig et al.) | PF, PI, DP (Dewitt et al.) |
| --- | --- | --- | --- |
| Baseline | 0 | 0 | 0 |
| 6 weeks | 0.144 | 0.099 | 0.09 |
| 12 weeks | 0.129 | 0.091 | 0.0906 |

Figure S1. Utility gain in all dimensions measured (PF=physical function, PI=pain interference, DP=depression, AX=anxiety) based on value sets from Dewitt et al. ^1^ (no value set for anxiety) and Craig et al ^2^.

**References**

1.	Dewitt B, Feeny D, Fischhoff B, et al. Estimation of a Preference-Based Summary Score for the Patient-Reported Outcomes Measurement Information System: The PROMIS((R))-Preference (PROPr) Scoring System. *Med Decis Making*. Aug 2018;38(6):683-698. doi:10.1177/0272989X18776637

2.	Craig BM, Reeve BB, Brown PM, et al. US valuation of health outcomes measured using the PROMIS-29. *Value Health*. Dec 2014;17(8):846-53. doi:10.1016/j.jval.2014.09.005
